# Supplementary figures and images for: Farm exposure is associated with human breast milk immune profile and microbiome
Source: bioRxiv. 2024 Oct 14:2024.10.14.618271. Preprint. [Version 1] doi: 10.1101/2024.10.14.618271 (PMC11507701; doi:10.1101/2024.10.14.618271)

Human breast milk IgA (mg/ml)

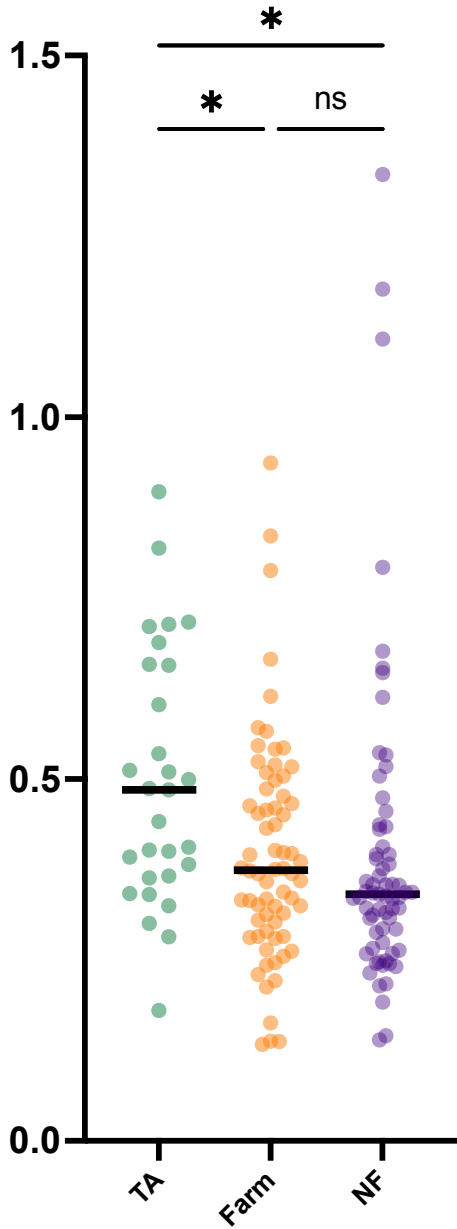

- TA
- Farm
- NF

Supplement: Supplement 2 — Supplemental Figure 1. Child farm score at the time of human breast milk collection (2 months), which is based on frequency of exposure with cattle & forage, goats, pigs, poultry, sheep, and horses (see Methods), is shown for traditional agrarian, farm, and non-farm mother-infant pairings (TA n=30, Farm n=63, Non-Farm n=59). [file media-2.pdf]

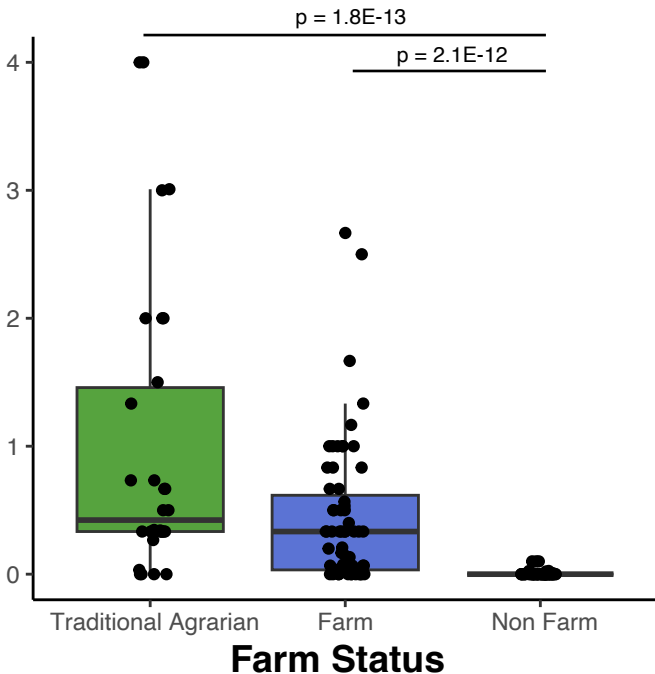

Supplement: Supplement 3 — Supplemental Figure 2. Breastmilk samples from TA mothers contain significantly increased IgA compared to Farm and Non-Farm groups. Breastmilk IgA levels at 2 months are shown compared to farming status.*p<0.05 [file media-3.pdf]

**A****Phylum**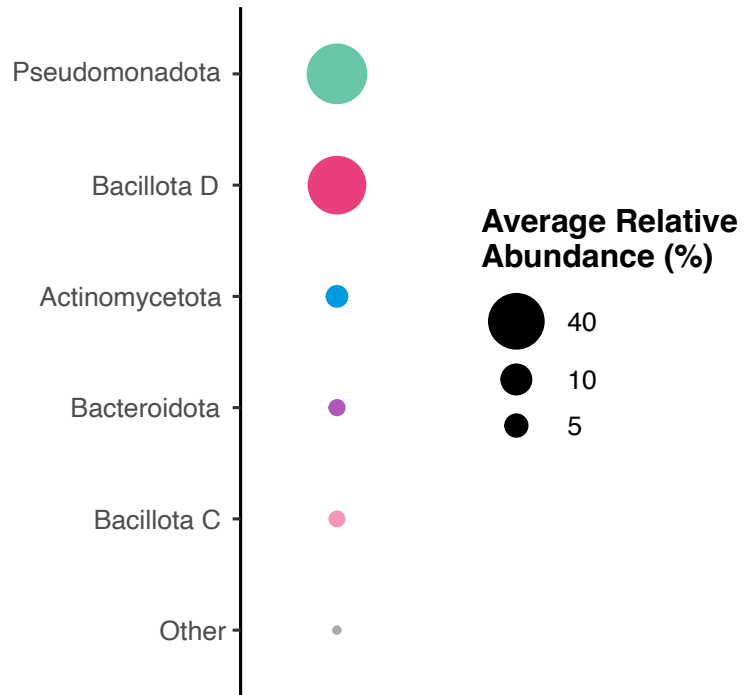**B****Genus**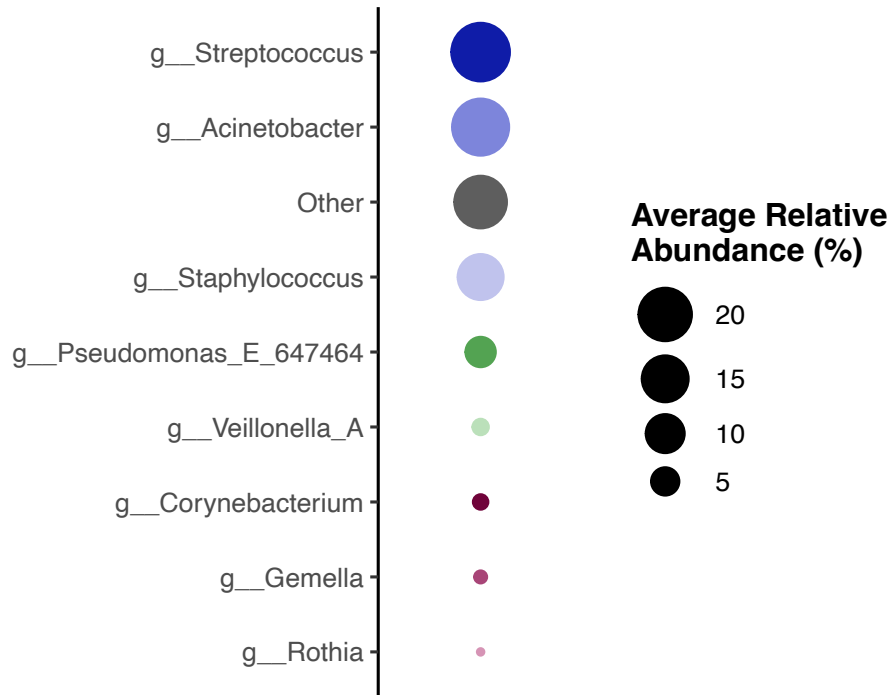

Supplement: Supplement 4 — Supplemental Figure 3. A) Average relative abundances of the top phyla present across all 149 human breast milk samples. Phlya present at an average of less than 1% relative abundance are grouped into “Other”. The Bacillota phylum is subdivided into groups A-D because it is polyphyletic within the Greengenes2 reference tree, which was used for Qiime2 taxonomic assignment. B) Average relative abundances of the top genera present across all human breast milk samples. Genera present at an average of less than 1% relative abundance are grouped into “Other”. [file media-4.pdf]

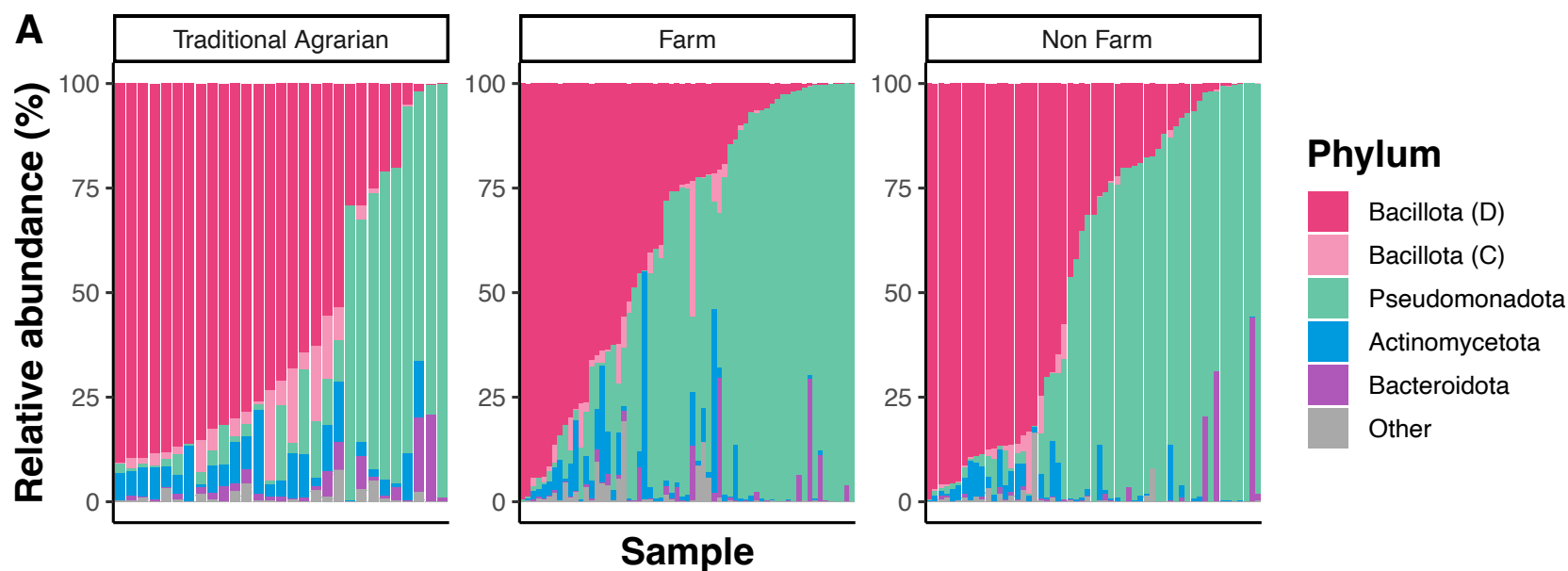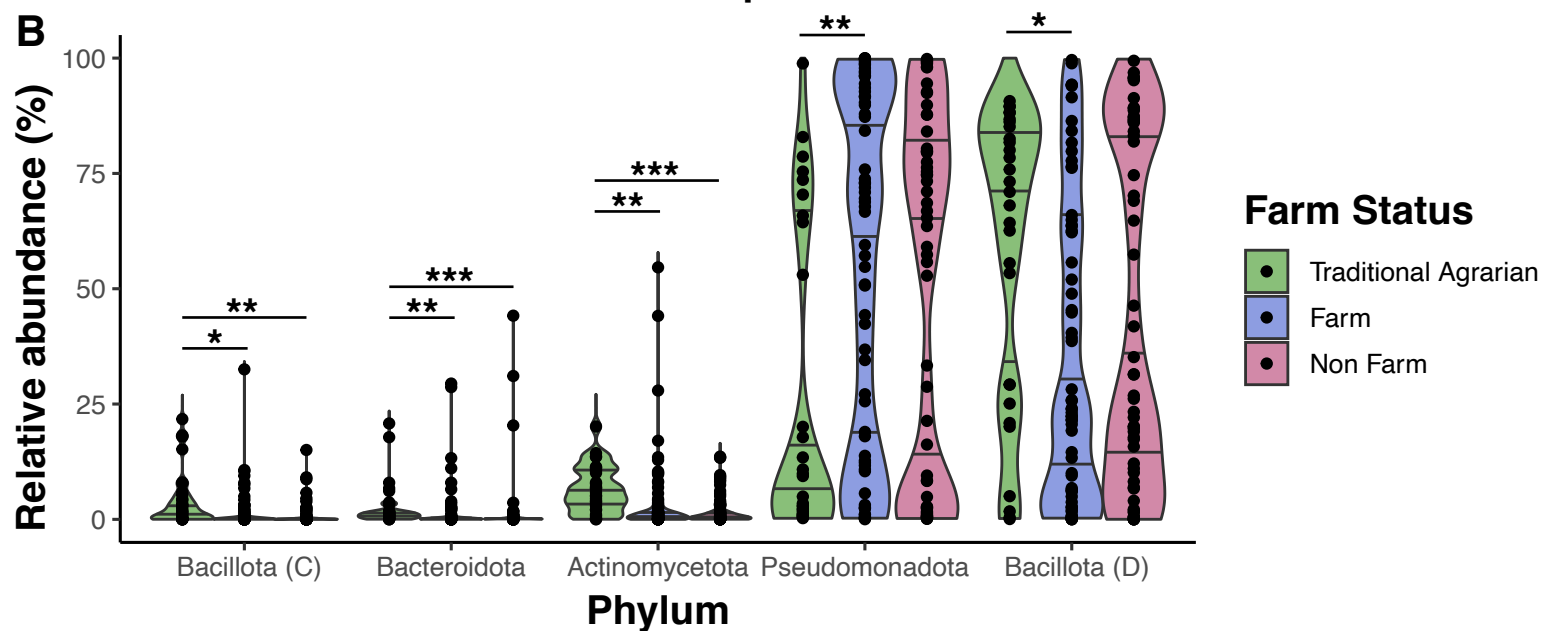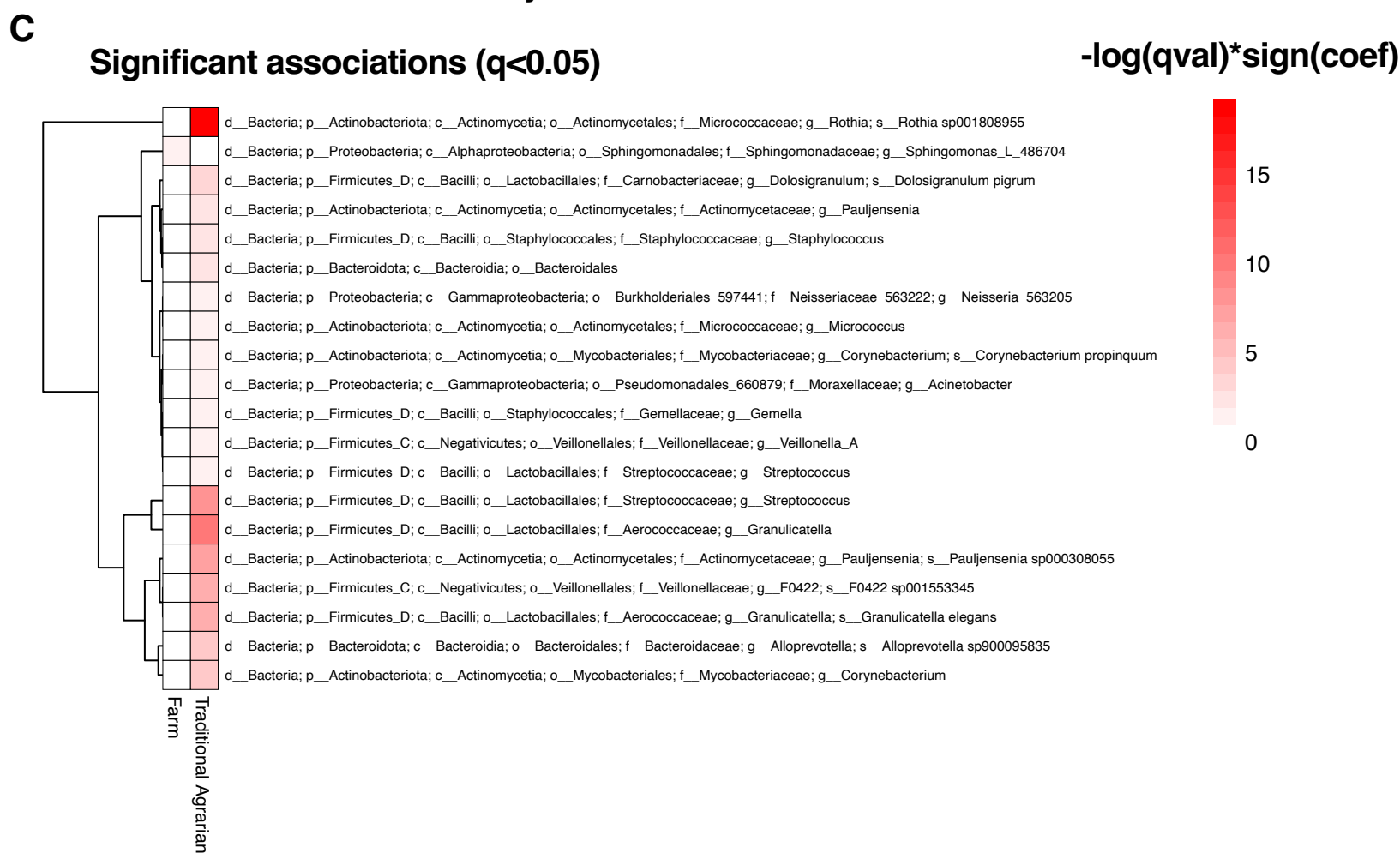

Supplement: Supplement 5 — Supplemental Figure 4. A) Microbial community composition of each sample based on relative abundance of ASVs at the phylum level. Samples are grouped by farm status and ordered by the Bacillota (D) phylum. B) Violin plot of Bacteroidota, Actinomycetota, Pseudomonadota, and Bacillota relative abundances in each human breast milk sample. *p<0.05, **p<0.01, ***p<0.001 based on a Kruskal-Wallis test, followed by a Dunn posthoc test with Benjamini-Hochberg p-value adjustment. C) Differentially abundant ASVs between farm/non-farm and traditional agrarian samples, with traditional agrarian as the reference group, determined using MaAsLin2. ASVs with a corrected q-value less than 0.05 are shown. Color within the heatmap takes into account q-value and sign of the coefficient (effect estimate), where a darker color indicates a larger difference between the test group (Farm or Non-Farm) and the reference group (Traditional Agrarian). [file media-5.pdf]
